# Adverse reaction to *Ficus Carica*: reported case of a possible cross-reactivity with Der p1

**DOI:** 10.1186/s12948-020-00125-6

**Published:** 2020-06-03

**Authors:** S. Urbani, A. Aruanno, E. Nucera

**Affiliations:** Allergy Unit, Fondazione Policlinico Universitario A. Gemelli IRCCS, Università Cattolica del Sacro Cuore, Largo F. Vito, 1, 00168 Rome, Italy

**Keywords:** Fig, Allergy, Cysteine proteases, Cross-reactivity

## Abstract

**Background:**

Ficus carica is an edible fruit, belonging to the Moraceae family, rarely described as cause of food allergy. We describe the first case of fig allergy that occurred as a cross-reactivity between fig and Derp 1.

**Case presentation:**

We present a case of a 10-years-old-girl, with a history of no-seasonal mild intermittent rhinitis, who experienced an immediate reaction after ingestion of a fresh fig. Skin prick tests (SPT) with commercial extracts of food, airborne allergens, latex and panallergens (profilin, PR-10 and lipid transfer protein) were performed. SPT revealed a sensitization only for dermatophagoides farina and dermatophagoides pteronyssinus which was then confirmed with by specific IgE assay (UniCAP, Phadia, Uppsala, Sweden). We also carried out a positive SPT with a commercial fig allergen (Lofarma, Milan, Italy) and prick-by-prick (PBP) both with skin and pulp of green raw and cooked fig. Fig specific serum IgE levels were 1.08 U/ml and specific IgE for rDer p1 was 16.20 U/ml (total serum IgE = 377 U/ml). In contrast specific IgE levels for latex, LTP, profilin, PR-10 and pollen allergens were negative.

**Conclusion:**

The ficin, the major fig allergen, belongs to cysteine protease family like Der p 1. The symptoms presented by our patient could be related to a cross reactivity between these two proteins which present a structural homology.

## Background

*Ficus carica* is an edible fruit, belonging to the Moraceae family, and rarely described as cause of food allergy. The cases of fig allergy reported in literature have been related to cross-sensitization to weeping fig (*Ficus benjamina*—FB), a common ornamental houseplant, or could be included in the context of “latex fruit syndrome” or “ficus-fruit syndrome” [1].

We describe the first case of fig allergy that occurred as a cross-reactivity between fig and dust mite proteins.

## Case presentation

We present a case of a 10-years-old-girl with a history of no-seasonal mild intermittent rhinitis, who experienced an immediate reaction characterized by an oral allergy syndrome (OAS), drooling, urticaria, lips and face angioedema and dyspnea after ingestion of a fresh fig. She was treated in emergency room with inhalator salbutamol and intravenous antihistamines and corticosteroids showing a progressive improvement of symptoms.

We performed a complete allergological work-up including skin prick tests (SPTs) (Lofarma, Milan, Italy; Alk-abellò, Lainate, Milan) with commercial extracts of latex, apple profilin, peach lipid transfer protein (LTP), other food and airborne allergens. We consider them positive when a wheal at least 3 mm greater in diameter than the negative control is observed. Furthermore, we confirmed SPTs results through total and specific serum assays (UniCAP, Phadia, Uppsala, Sweden), considering positive specific IgE values greater than 0.35 kU/L.

**Fig. 1:**
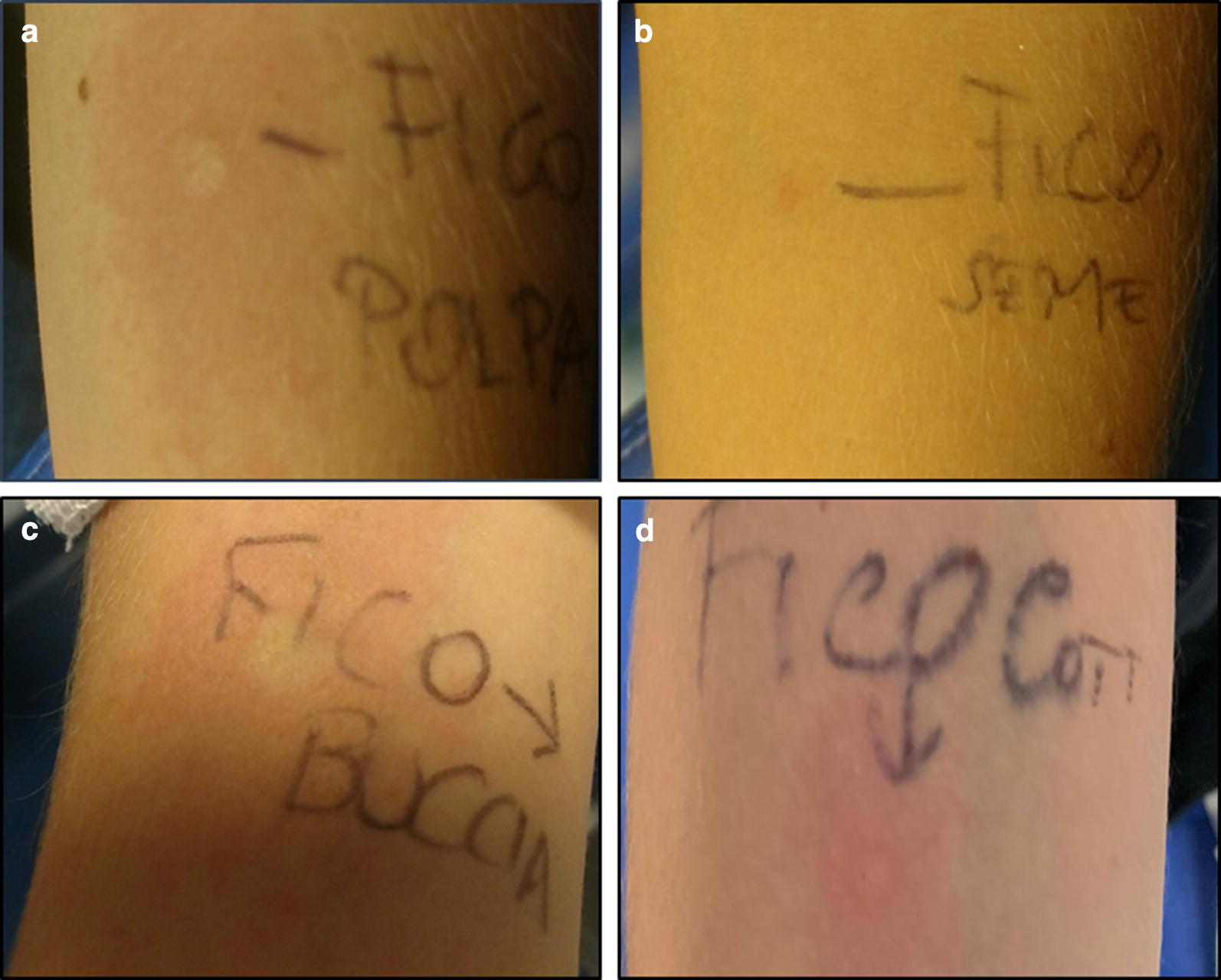
Prick by prick with raw fig seed (**a**), pulp (**b**) and peel (**c**) and with cooked fig (**d**)

For our patient, SPTs revealed only a dermatophagoides sensitization, which was then confirmed by specific Der p 1 and Der p 2 IgE assays (Table 1). We also carried out a positive SPTs with a commercial fig allergen (Lofarma, Milan, Italy). The prick-by-prick (PBP) performed with peel and pulp of raw and cooked fig resulted positive as well, while PBP with fig seed showed negative results (Fig. 1). Suspecting a Ficus-Fruit-Syndrome, also PBP for kiwi, avocado, papaya, banana and pineapple were performed with negative results.

**Table 1:** Summary of the main patient laboristic and cutaneous tests

Allergen	Mean wheal size of SPT (mm)	Specific IgE (U/ml)
Fig		
Commercial extract	4	1.08
Cooked		
Pulp	3	
Peel	3	
Raw		
Pulp	6	
Peel	5	
Seed	0	
Dermatophagoides pteronyssinus		
Der p 1	8	16.20
Der p 2		37.70
Farinae	7	/

Fig specific serum IgE levels were 1.08 U/ml (total serum IgE 377 U/ml), while sIgE levels for latex, Bet v 1, nsLTP, profilins, PR-10 proteins and pollens were negative.

Although our patient refused an oral placebo-controlled challenge with the fig, the diagnosis of fig allergy is very probable on the basis of clinical history and allergological work-out.

## Discussion

Figs are rarely been reported as a primary cause of food allergy; therefore, in the diagnosis of this allergy, it is always necessary considering the cross-reactivity reported to date.

Adverse reactions to fig could be due to four main characterized allergens: Fig c 4—profiline [2], Fig c Ficin—protease [3], Fig c—Lipid Transfer protein [4] and a 17 kDa protein, a Bet v 1 homologue [5].

Additionally, in most cases, fig reactions have often been described in the context of “latex-fruit syndrome”, an allergic disease resulting from cross-sensitization to latex (Hevea brasiliensis) and several types of fruits [6]. In this case, the involved allergens seem to be proteins that cross-react with hevein-like domains of Ficus.

Moreover, figs can cause allergic reactions in subjects sensitized to FB allergens independent of sensitization to rubber latex allergens. The FB allergen is airborne and the sensitization occurs through inhalation. Indeed, almost 90% of patients with allergic reactions to this fruit had a weeping fig in their home [7]. These data suggest that, in some cases, fig allergy could be a consequence of primary inhalant sensitization to FB latex proteins and the perseverance of cross-reactive proteins in fig fruits, similarly to pollen-associated food allergy [8].

In our case we have excluded these sensitizations considering that the patient had never been exposed to FB and she didn’t experience an adverse reaction after latex exposure.

We have reported a case of OAS followed by systemic respiratory and cutaneous symptoms after ingestion of fig, similar to the two cases described by Antico et al. [9]. It is worth noting that the patients of Antico et al. [9] show fig and grass/birch pollen sensitization without being sensitized to weeping fig or having the latex-fruit syndrome. Then, in these cases the main allergic manifestation (OAS) and the positivity of SPTs pollen and fig peel allow us to hypothesize that the involved allergens are related to labile panallergens cross-reacting to pollens.

In fact, Hemmer et al. [2] demonstrate the presence of PR-10 allergens in Moraceae fruits with an high prevalence of cross-reactivity with Bet v 1 and they provide a first-time evidence for a close relationship between birch pollen sensitization and fig allergy. The proven presence of homologous allergens of PR10 (Bet v 1-like) in fig and other Moraceae has recently provided a molecular basis to food allergy associated with birch pollen sensitization. This fact could explain the absence of symptoms after ingestion of dried figs in these patients, due probably to the proteolysis of PR10 proteins during the drying process [2].

Anyway, in our case, specific IgE assay denied the presence of a simultaneously sensitization to grass and birch pollens; this ruled out that fig adverse reaction is due to aforesaid pollen cross-reactivity.

In most cases, however, the ficin belonging to the group of cysteine proteases appears to be the major allergen involved in ficus-associated reactions. This group also includes the major allergens of some fruits, such as the actinidin (Act d 1) of kiwi, the papain (Car p 1) of papaya and the bromelain (Ana c 2) of pineapple, but also one of major allergens of dust mites (Der p 1), which seem to have a partial homology with these fruit proteins.

Since Der p 1 belong to the same molecular family of ficin, we hypothesize that our patient fig adverse reaction was due to this cross reactivity.

Furthermore, due to cysteine proteases cross-reacting, we recommend to avoid the ingestion of kiwi, papaya, pineapple and mulberry. The latter is included because in a case series of Caiaffa et al. [10], the authors described 3 cases of associated fig and mulberry allergy. Although we did not carry out any allergic work-up for mulberry, for difficulties in finding the extract or the fresh fruit, we advise the patient to not ingest this fruit.

## Conclusion

In summary, upon suspicion of a fig allergy, an accurate clinical history and a deep allergological evaluation are very important in order to correctly address the molecular origin of adverse reaction.

## Data Availability

Not applicable.

## Abbreviations

FB: *Ficus benjamina*
OAS: Oral allergy syndrome
SPTs: Skin prick tests
LTP: Lipid transfer protein
